# Estimation of pulmonary vascular resistance for Glenn physiology

**DOI:** 10.1371/journal.pone.0307890

**Published:** 2024-07-26

**Authors:** Sebastian Laudenschlager, Samuel Schofield, Nicolas Drysdale, Matthew Stone, Jennifer Romanowicz, Benjamin Frank, Michael DiMaria, Vitaly O. Kheyfets, Mehdi Hedjazi-Moghari

**Affiliations:** 1 Department of Radiology, School of Medicine, University of Colorado, Aurora, CO, United States of America; 2 Department of Cardiology, University of Colorado and Children’s Hospital Colorado, Aurora, CO, United States of America; 3 Department of Surgery, School of Medicine, University of Colorado, Aurora, CO, United States of America; 4 Department of Surgery, University of Colorado and Children’s Hospital Colorado, Aurora, CO, United States of America; 5 Department of Critical Care, University of Colorado and Children’s Hospital Colorado, Aurora, CO, United States of America; 6 Department of Radiology, University of Colorado and Children’s Hospital Colorado, Aurora, CO, United States of America; University of Southern California, UNITED STATES OF AMERICA

## Abstract

Children with single ventricle heart disease typically require a series of three operations, (1) Norwood, (2) Glenn, and (3) Fontan, which ultimately results in complete separation of the pulmonary and systemic circuits to improve pulmonary/systemic circulation. In the last stage, the Fontan operation, the inferior vena cava (IVC) is connected to the pulmonary arteries (PAs), allowing the remainder of deoxygenated blood to passively flow to the pulmonary circuit. It is hypothesized that optimizing the Fontan anatomy would lead to decreased power loss and more balanced hepatic flow distribution. One approach to optimizing the geometry is to create a patient-specific digital twin to simulate various configurations of the Fontan conduit, which requires a computational model of the proximal PA anatomy and resistance, as well as the distal Pulmonary Vascular Resistance (PVR), at the Glenn stage. To that end, an optimization pipeline was developed using 3D computational fluid dynamics (CFD) and 0D lumped parameter (LP) simulations to iteratively refine the PVR of each lung by minimizing the simulated flow and pressure error relative to patients’ cardiac magnetic resonance (CMR) and catheterization (CATH) data. While the PVR can also be estimated directly by computing the ratio of pressure gradients and flow from CATH and CMR data, the computational approach can separately identify the different components of PVR along the Glenn pathway, allowing for a more detailed depiction of the Glenn vasculature. Results indicate good correlation between the optimized PVR of the CFD and LP models (n = 16), with an intraclass correlation coefficient (ICC) of 0.998 (p = 0.976) and 0.991 (p = 0.943) for the left and right lung, respectively. Furthermore, compared to CMR flow and CATH pressure data, the optimized PVR estimates result in mean outlet flow and pressure errors of less than 5%. The optimized PVR estimates also agree well with the computed PVR estimates from CATH pressure and CMR flow for both lungs, yielding a mean difference of less than 4%.

## 1. Introduction

Staged surgical palliation is a treatment approach for patients with single-ventricle heart disease that aims to establish a sequential arrangement of the systemic and pulmonary circulations, utilizing the single ventricle to pump blood into the systemic circulation [1]. This process involves a series of surgeries, namely: (1) the Norwood operation, (2) the Glenn (either bidirectional Glenn or hemi-Fontan) procedure, and (3) the extracardiac Fontan completion, ultimately leading to the creation of a total cavopulmonary connection (TCPC). In this connection, the inferior vena cava (IVC) and superior vena cava (SVC) are joined to the right and left pulmonary arteries (RPA and LPA) respectively, bypassing the heart [2].

Even with the TCPC, patients continue to experience long-term complications, some of which are associated with the hemodynamic performance of the TCPC design [3, 4]. For example, the increased power losses (PL) at the TCPC connection have been linked to reduced exercise capacity in patients with single-ventricle conditions [5]. Furthermore, an imbalanced distribution of hepatic flow between the left and right lungs may contribute to the formation of pulmonary arteriovenous malformations [6, 7]. Therefore, it is crucial to ensure an optimal design for the TCPC connection that minimizes power losses (PL) and achieves a balanced hepatic flow distribution (HFD) to both the left and right lungs. Previous studies have primarily concentrated on enhancing hemodynamic efficiency by not only reducing PL through the implementation of extracardiac Fontan conduits but also enhancing the HFD [8–11]. These studies have utilized computational fluid dynamics (CFD) to investigate the hemodynamic outcomes of modified extracardiac Fontan conduits [12, 13]. However, they have not specifically modeled extracardiac Fontan conduit structures in single-ventricle patients with Glenn physiology, their focus has primarily been on revising and modifying existing extracardiac Fontan structures in order to reduce PL and achieve a more balanced hepatic flow distribution (HFD) [14].

Accurately estimating the pulmonary vascular resistance (PVR) of each lung in children with single ventricle conditions is crucial for simulating the Fontan operation. By determining the PVR of each lung during the Glenn stage, it becomes possible to estimate the blood flow to the left and right lungs during the Fontan stage. Unfortunately, individual lung PVR values are not routinely measured in clinical practice, and only the total body mass indexed PVR is calculated through invasive catheterization (CATH) [15]. In addition, this CATH-based PVR calculation relies on Fick-based estimations of cardiac output, which can be inaccurate and can result in significantly different pulmonary flow than flow measured by cardiovascular magnetic resonance (CMR) imaging [16, 17]. The PVR of each lung can also be computed directly via CATH pressure and CMR flow, but this depends on physiological pressure gradients from CATH, which may not always be reliable, and provides only a single PVR value for each lung and is thus unable to provide information on the differences in resistance along the Glenn pathway.

In this manuscript, we present a novel and computationally inexpensive method for estimating the PVR of each individual lung along with the resistances of the Glenn pathway. Our approach utilizes either 3D CFD models or 0D lumped parameter (LP) models, with pressure measurements obtained from CATH and anatomical and flow data derived from CMR. This approach has the advantage of characterizing the entire flow circuit along the Glenn pathway, which can potentially offer surgical guidance for the Fontan procedure to optimize post-surgical blood flow distribution. To assess the two computational models, we conducted a comparison between their resulting flow, pressure, and estimated PVR. For validation, we compared the flow, pressure, and estimated PVR to the measured values obtained via CMR and CATH.

## 2. Methods

### 2.1. Patient demographics

The patient dataset comes from a retrospectively identified and anonymized patient dataset gathered over the past ten years and accessed from January 2023 to March 2024 for analysis. This retrospective study was approved by the Colorado Multiple Institutional Review Board (IRB 19–1420) and informed consent was waived. The dataset consists of 16 children (10 male, 6 female, median age = 4 years, age range 2–5) with Glenn physiology (13 bidirectional Glenn, 3 hemi-Fontan), who were referred to Children’s Hospital Colorado and underwent a CMR exam and a diagnostic CATH. Most patients had the CMR and CATH exam on the same day or within the same week (n = 14), but in two cases, the exams were separated in time by approximately 6 months and a year, respectively. The CMR images were used to evaluate the morphology of the Glenn pathway and generate a 3D model of this pathway. Additionally, the CMR images were used to assess the blood flow in the superior vena cava (SVC), the left pulmonary artery (LPA), and the right pulmonary artery (RPA) by using a phase-contrast spoiled gradient echo sequence. Because the CMR flow data represents averaged flow, respiration is not considered. The CATH exam was used to collect the mean pressure values at the SVC, LPA, RPA, left atrium (LA), and right atrium (RA), in addition to pulmonary flow (*Q*_*p*_) and total PVR. Table 1 displays the patients’ demographics along with their CMR and CATH measurements. Utilizing the clinical data from CMR and CATH exams, a 3D CFD model and a 0D LP model were developed for each patient.

**Table 1:** Demographic and hemodynamic data for the patient dataset used for this study. All flow rates given are indexed by patient-specific BSA.

SUBJECTS				CMR			CATH						
**PID**	Sex	BSA	Age	*Q*_*SVC*_	*Q*_*LPA*_	*Q*_*RPA*_	PVR	*Q*_*p*_	*P*_*SVC*_	*P*_*LPA*_	*P*_*RPA*_	*P_LA_*	*P_RA_*
		(*m*^2^)	(years)	*(L/min/m* ^*2*^ *)*	*(L/min/m* ^*2*^ *)*	*(L/min/m* ^*2*^ *)*	(*WU*∙*m*^2^)	*(L/min/m* ^*2*^ *)*	*(mmHg)*	*(mmHg)*	*(mmHg)*	*(mmHg)*	*(mmHg)*
**1**	M	0.64	4	1.71	0.65	1.13	1.87	2.14	11	9	10	5	5
**2**	M	0.66	5	1.31	0.66	0.63	1.38	2.9	7	7	7	3	3
**3**	M	0.6	5	1.27	0.63	0.65	3.8	1.84	12	12	12	5	4
**4**	F	0.51	4	2.31	1.12	1.22	2.21	2.26	9	9	8	4	4
**5**	F	0.55	4	1.85	0.65	1.20	2.18	3.22	11	11	11	4	4
**6**	F	0.63	5	1.23	0.52	0.70	2.04	2.93	10	10	10	4	4
**7**	M	0.67	5	1.92	1.05	0.90	1.22	2.47	7	7	7	4	4
**8**	F	0.57	5	1.49	0.80	0.70	1.72	2.33	8	8	8	4	4
**9**	M	0.52	3	1.65	0.74	0.86	2.81	1.78	9	8	9	4	3
**10**	M	0.49	4	3.13	1.98	1.14	1.4	2.14	9	8	8	5	5
**11**	M	0.54	3	2.02	0.55	1.44	1.3	3.07	11	11	11	7	5
**12**	F	0.55	3	2.16	1.36	0.77	1.12	2.67	8	8	7	4	4
**13**	M	0.53	3	1.81	0.58	1.22	1.63	2.45	8	7	8	3	3
**14**	M	0.7	4	2.17	0.98	1.15	2.12	1.89	9	8	9	5	5
**15**	F	0.49	3	2.90	1.45	1.50	1.37	3.64	10	10	10	5	5
**16**	M	0.31	2	2.60	1.84	0.77	0.68	5.89	10	9	9	5	6
**Mean±SD**		**0.56±0.09**	**3.88±0.96**	**1.97±0.56**	**0.97±0.46**	**1.00±0.28**	**1.80±0.75**	**2.73±1.00**	**9.31±1.49**	**8.88±1.54**	**9.00±1.55**	**4.44±0.96**	**4.25±0.86**

### 2.2. CFD (3D) model

Blood flow on a three-dimensional domain Ω^3*D*^ is modeled using the incompressible Navier-Stokes equations

$$\rho \left(\frac{\partial u}{\partial t}+u∙\nabla u\right)-\nabla ∙\tau =\rho f,$$

$$\nabla ∙\tau =-pI+\mu (\nabla u+\nabla u^{T}),$$ (1)

∇∙u=0,

where ***u***(***x***, *t*) and *p*(***x***, *t*) represent the velocity and pressure fields (***x***∈Ω^3*D*^, *t*∈ℝ_≥0_), respectively, of blood with density *ρ* and dynamic viscosity *μ*, subject to an external force ***f***(***x***, *t*) and a stress tensor ***τ***(***x***, *t*) (***I*** is the identity matrix). The equations were solved computationally using SimVascular’s svSolver, an open-source finite element implementation [18].

**Fig 1:**
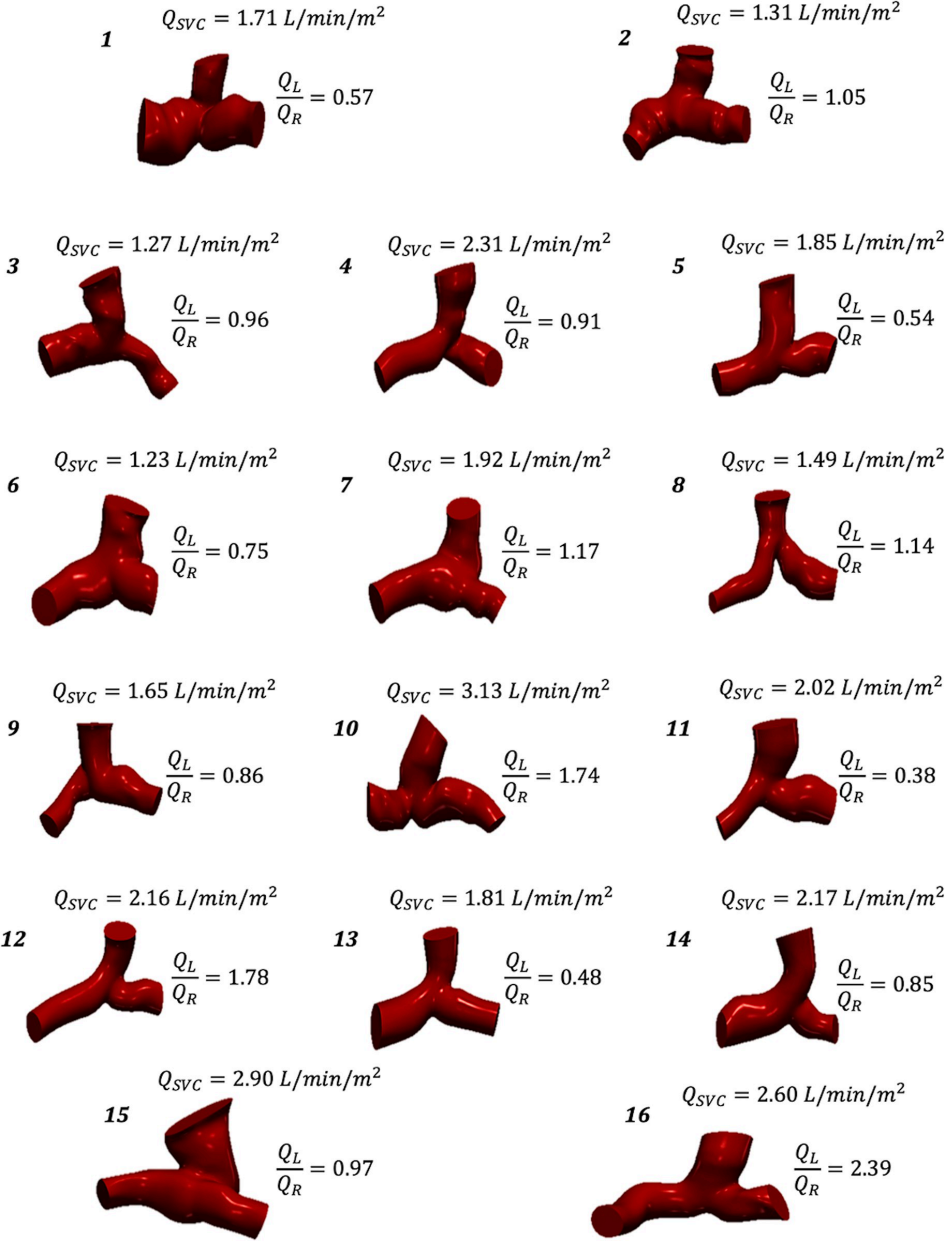
3D models of the Glenn PA junction for all 16 patients, along with CMR measured indexed inlet flow rates (*Q*_*SVC*_), along with the ratio of LPA flow rates (*Q*_*L*_) *and RPA flow rates* (*Q*_*R*_).

The patient-specific CFD models, which model the SVC and pulmonary artery (PA) junction, were created by manually segmenting the clinically acquired CMR images using the SimVascular segmentation module [18]. The segmentation process consisted of identifying the SVC, LPA, and RPA on the CMR image stack, creating a path and corresponding 2D contours for each vessel using splines, lofting each vessel into a 3D model, and performing a union of the vessels to combine them into a single 3D model. The models were subsequently cleaned up in a post-processing stage with smoothing and blending. This segmentation procedure is similar to that found in previous studies [14], and the resulting patient models are shown in Fig 1.

Once the 3D models have been created, they were used to create finite element meshes using TetGen [19]. The finite element meshes for all patients are unstructured with an upper bound on edge length set to 1.5 mm, resulting in meshes with approximately 15-50k elements, depending on the vessel morphology. To ensure mesh independence, the flow and pressure solutions using the 1.5mm meshes were compared with those of an equivalent reference mesh consisting of approximately 3 million elements. The flow and pressure solutions using the 1.5mm mesh were within 1% of those of the 3 million element mesh.

**Fig 2:**
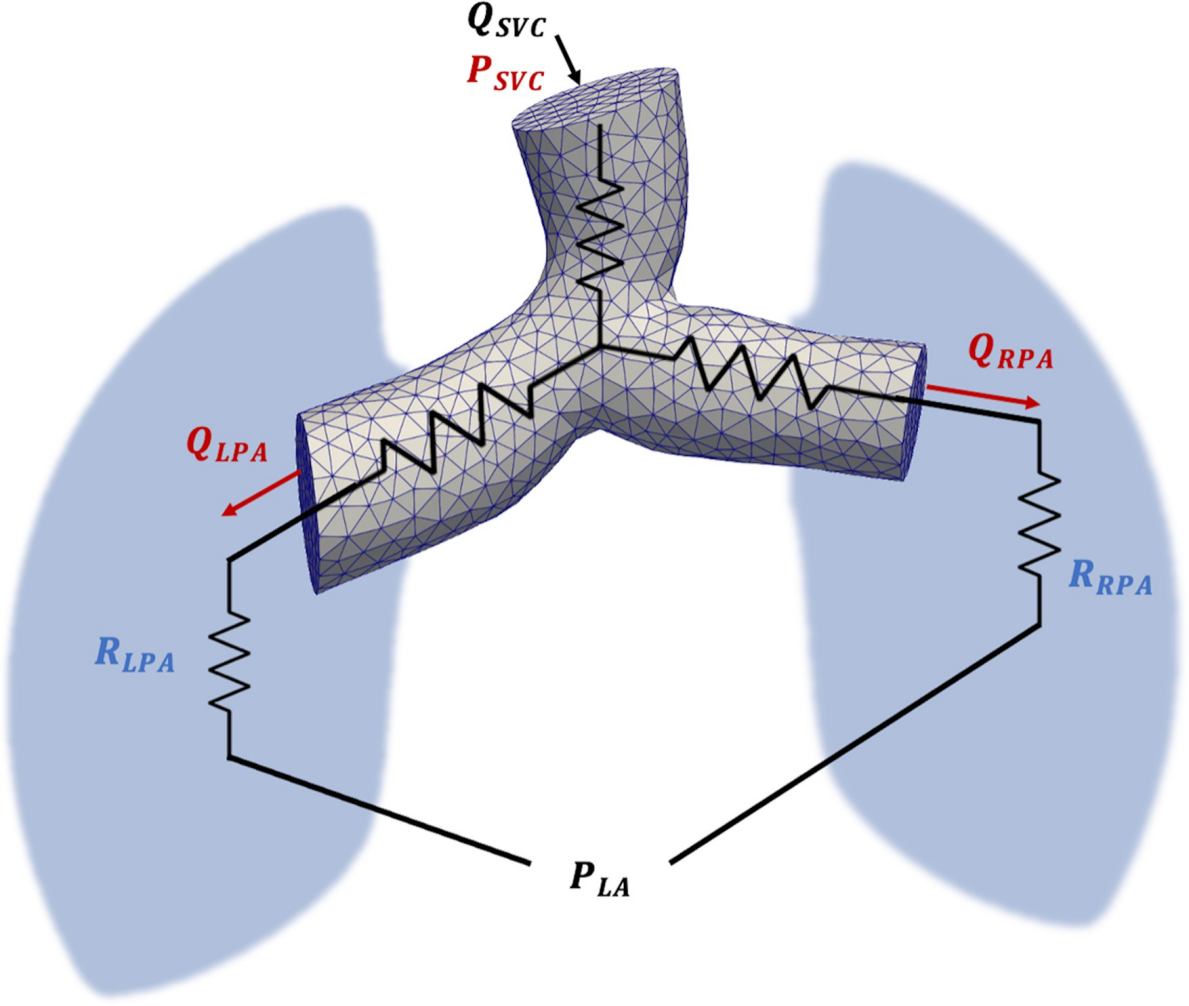
PA model overview at the Glenn stage. Each simulation uses inlet flow and outlet resistances as model inputs. For PVR optimization, the outlet resistances are iteratively optimized over a series of simulations, by minimizing a cost function comprised of inlet pressure error and outlet flow errors. Fixed quantities are indicated in black, optimization target variables are indicated in blue, and quantities used in the cost function evaluation are indicated in red.

Two types of boundary conditions were used for the inlets and outlets of the CFD model: (1) the inlet surface (SVC) uses a Dirichlet boundary condition specifying the inlet flow profile, acquired from CMR data and unique to each patient, and (2) the outlet surfaces (LPA, RPA) use a resistive boundary condition to capture the viscous effects of the downstream vasculature, as shown in Fig 2. On the walls of the domain, which are rigid for all CFD simulations, a no-slip boundary condition is enforced. All CFD simulations were run in parallel with 4 processers and a timestep of 0.001 s. Additionally, all CFD simulations were run for three cycles to ensure results have converged to a periodic solution. Blood in the CFD simulations was assumed to be Newtonian, a reasonable assumption in larger vessels such as the pulmonary artery [20, 21], with a constant fluid viscosity of *μ* = 0.04 poise and a constant fluid density of *ρ* = 1.06 *g*∙*cm*^−3^.

### 2.3. LP (0D) model

The 0D LP models simulate only bulk flow and pressure by using electrical circuit analogues (current and voltage), ignoring patient-specific vessel morphology. These models aim to produce an approximation of the flow and pressure in the CFD models, while being significantly less computationally expensive. The LP models were created using MATLAB’s Simulink [22] package, where each LP model starts with the same inlet flow as its CFD equivalent, as shown in Fig 2. Every vessel (including the PA junction) is represented by a model resistor, while outlet boundary conditions are represented by a resistor and a constant voltage analogous to distal pressure in the CFD model. To match the CFD model, compliance was not included in the LP model. Thus, each element of the 0D model is governed simply by the equation

ΔP=RQ, (2)

where Δ*P* is the pressure gradient, *R* is the resistance, and *Q* is the flow. Due to the simplicity of this model and the periodicity of the inlet flow profile, steady-state convergence is immediate, requiring only a single cycle per simulation.

### 2.4. PVR estimation

Fig 2 displays a schematic diagram of the PVR estimation pipeline. To optimize PVR estimation, we developed an optimization pipeline that iteratively performs CFD and LP simulations until simulated flow and pressure data matches, as closely as possible, clinically acquired data for each patient. For both CFD and LP models, two parameters are optimized: *R*_*LPA*_ and *R*_*RPA*_. The optimization uses iterative simulations to minimize a cost function that quantifies the error between simulation and clinical data. Given a cardiac cycle length *l* and a timestep Δ*t*, we define $n=\frac{l}{\Delta t}$ as the number of timesteps in a cardiac cycle. To match the temporal discretization, the CMR-measured flow was linearly interpolated, enabling a direct comparison between clinical and simulated flow results. The cost function was defined as

$$f(t)=\frac{1}{n}\sum_{t=0}^{n}\frac{{\left(Q_{LPA}^{c}(t)-Q_{LPA}^{s}(t)\right)}^{2}}{{\bar{Q}}_{LPA}^{c}}+\frac{{\left(Q_{RPA}^{c}(t)-Q_{RPA}^{s}(t)\right)}^{2}}{{\bar{Q}}_{RPA}^{c}}+\frac{{\left({\bar{P}}_{SVC}^{c}-{\bar{P}}_{SVC}^{s}\right)}^{2}}{{\bar{P}}_{SVC}^{c}}$$ (3)

where the superscripts *c* and *s* denote the clinically measured and simulated data, respectively, while $\bar{Q}$ and $\bar{P}$ represent time-averaged flow rates and pressure over a cardiac cycle. To minimize the objective function, we used the Nelder-Mead simplex method [23] with a tolerance of 1e-4. The initial resistances from which the optimization begins are computed via mean flow and pressure data from CMR and CATH and averaged across all patients, so that each patient’s PVR optimization starts from the same pair of outlet resistances.

### 2.5. Statistical analysis

To test the PVR estimation algorithm, the optimization pipeline was run on all 16 patients using both the CFD and LP model. The result of each optimization consists of a pair of resistances for the left and right lung, and simulation output data (flow and pressure) using the optimized PVR combination. From this, the estimated PVR can be further decomposed into vascular and lung resistance components. The estimated PVR for each lung and the simulated flow and pressure data for both models are then compared to each other and to clinical measurements in order to determine the viability of using CFD and LP models for PVR estimation. Additionally, the pulmonary blood flow measured from CMR exam was compared with the ones calculated from Fick’s principle [24] in CATH exam, and the estimated total PVR using CFD model was compared with the total PVR measured by CATH.

Descriptive statistics are reported as median and range, or mean ± standard deviation, as appropriate. Normality was tested using the Shapiro-Wilk test [25]. Either a two tailed paired t-test or a Wilcoxon signed-rank test [26] were used to compare two groups of paired data with Gaussian and non-Gaussian distributions, respectively. A p-value ≤ 0.05 was considered statistically significant. To assess agreement, Bland-Altman analysis [27] and intraclass correlation coefficient (ICC) [28] were used.

## 3. Results

**Fig 3:**
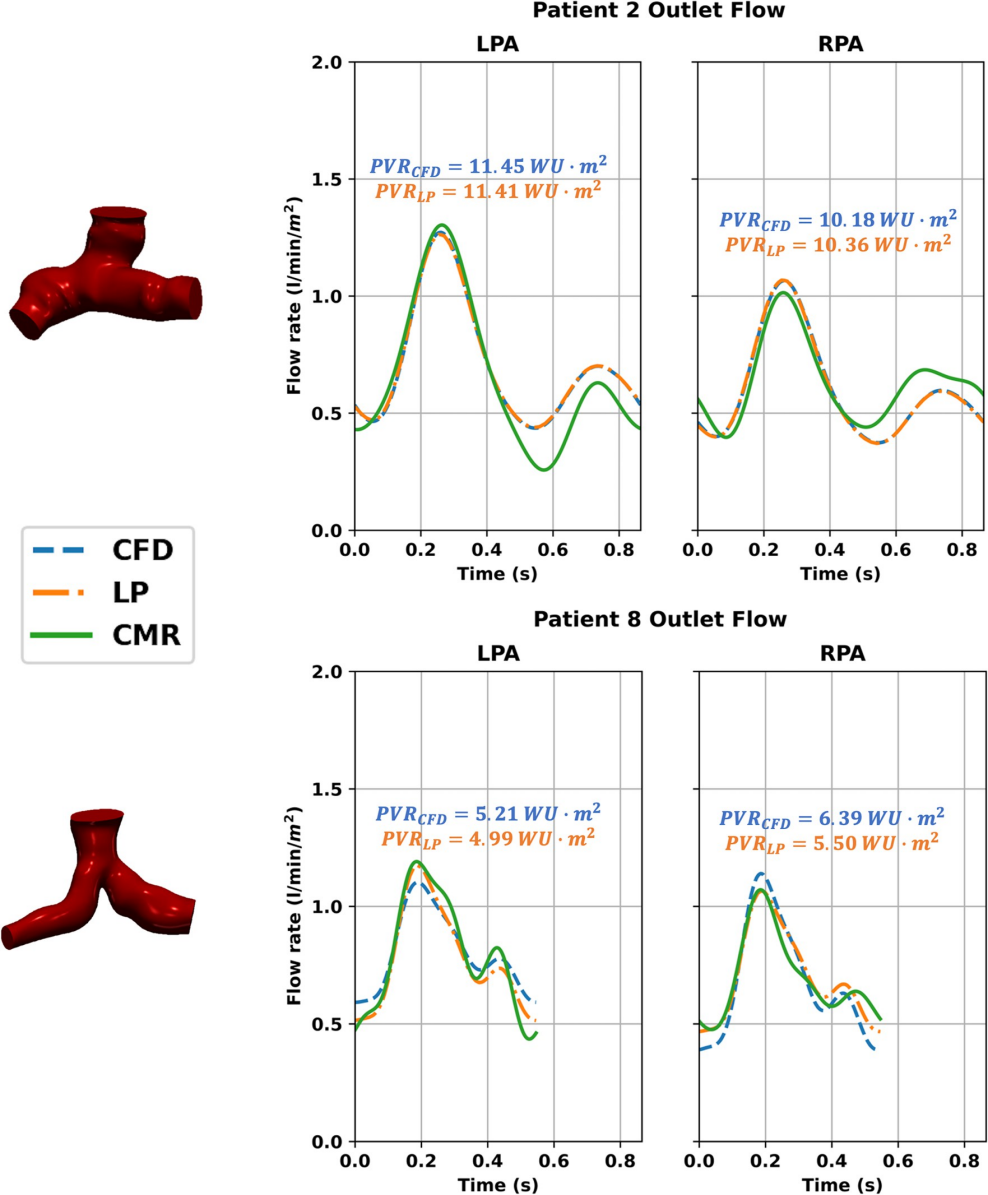
Indexed outlet flow curves for two patients over one cardiac cycle, representing two cases where there is good agreement (top) and noticeable disagreement (bottom) between the CFD and LP flow curves.

In the CFD model, pressure at the inlet and outlet surfaces is computed as a mean pressure across the entire surface, giving a single averaged pressure value at each timestep. Since only a single clinically measured value for pressure at each inlet/outlet was available, the computed pressure from the CFD model is further averaged over a cardiac cycle to allow for comparison to the CATH-based pressure. Similarly, the outlet pressure in the LP model is also averaged over a cardiac cycle. For both models, flow data consists of outlet waveforms over the same cardiac cycle as the CMR flow. For analysis, these flow curves are then summarized by time-averaged flow rates, which are indexed by patient body surface area (BSA). The simulated flow and pressure data for the CFD and LP models was then compared to determine the viability of using LP models for PVR estimation. Fig 3 shows two examples of the comparison between indexed CFD, LP, and CMR flow curves, indicating close agreement between the CFD and LP models.

### 3.1. Flow and pressure

**Fig 4:**
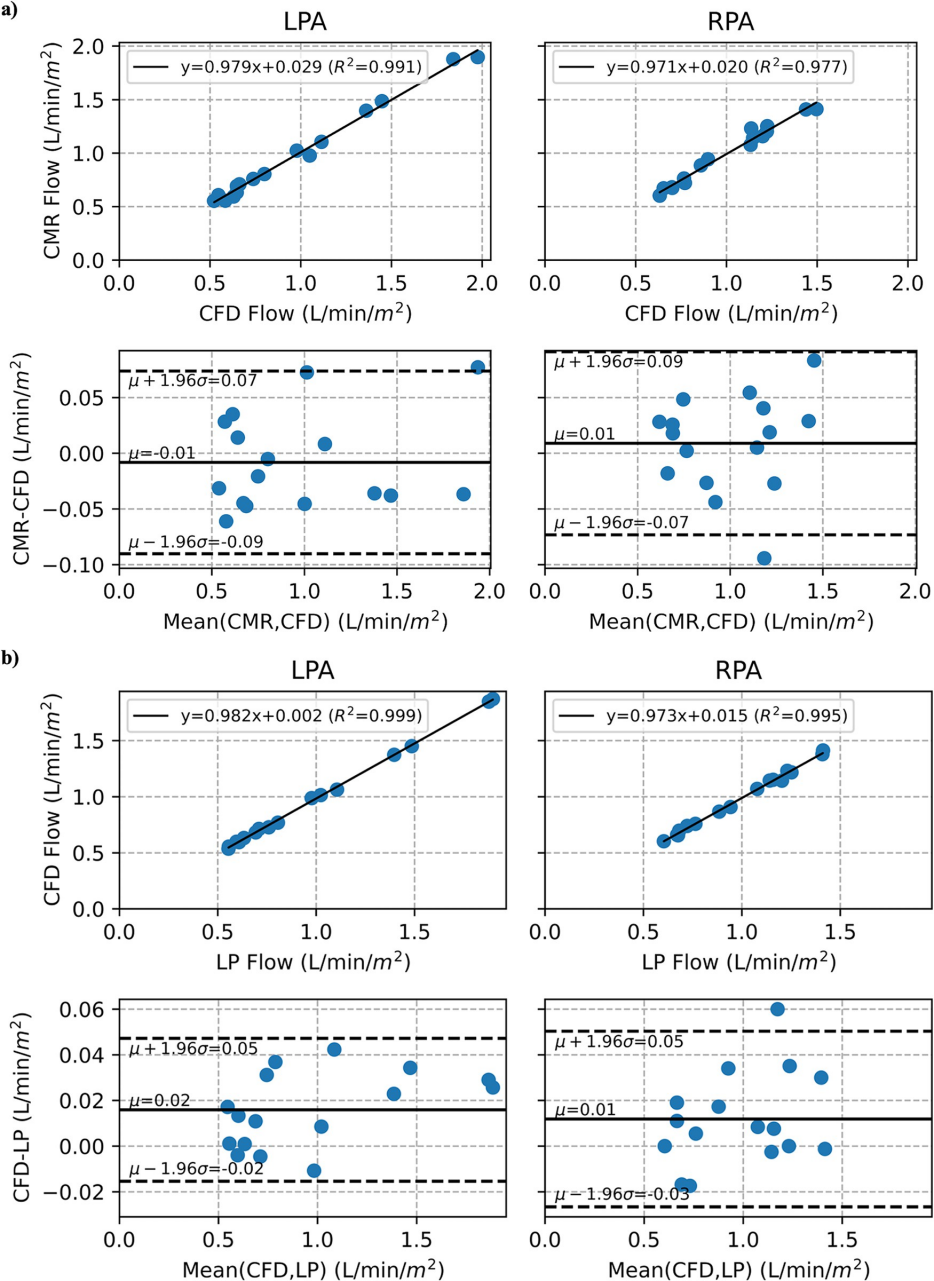
Mean indexed outlet flow comparison between (a) CMR and CFD, and (b) CFD and LP.

When comparing mean indexed outlet flow of the CFD and LP optimized models, we found excellent agreement for both the LPA (CFD 0.980 ± 0.454 L/min/m^2^ vs. LP 0.964 ± 0.447 L/min/m^2^; p-value = 0.921; ICC = 0.999 [0.99–1.00]) and the RPA (CFD 0.989 ± 0.279 L/min/m^2^ vs. LP 0.977 ± 0.272 L/min/m^2^; p-value = 0.904; ICC = 0.997 [0.99–1.00]). Relative to CMR-measured indexed blood flow (LPA 0.971 ± 0.462 L/min/m^2^, RPA 0.998 ± 0.284 L/min/m^2^), we find the CFD and LP models to have the comparable accuracy, both in the LPA (CMR vs. CFD; p-value = 0.960; ICC = 0.996 [0.99–1.00] and CMR vs. LP; p-value = 0.962; ICC = 0.996 [0.99–1.00]) and the RPA (CMR vs. CFD; p-value = 0.929; ICC = 0.988 [0.97–1.00] and CMR vs. LP; p-value = 0.834; ICC = 0.986 [0.96–1.0]). Fig 4 shows the linear regression and Bland-Altman plots of the mean indexed LPA and RPA outlet flows of the CFD model compared to CMR indexed blood flow, as well as that of the CFD and LP models, indicating good agreement in indexed outlet blood flow between the two models and CMR data.

**Fig 5:**
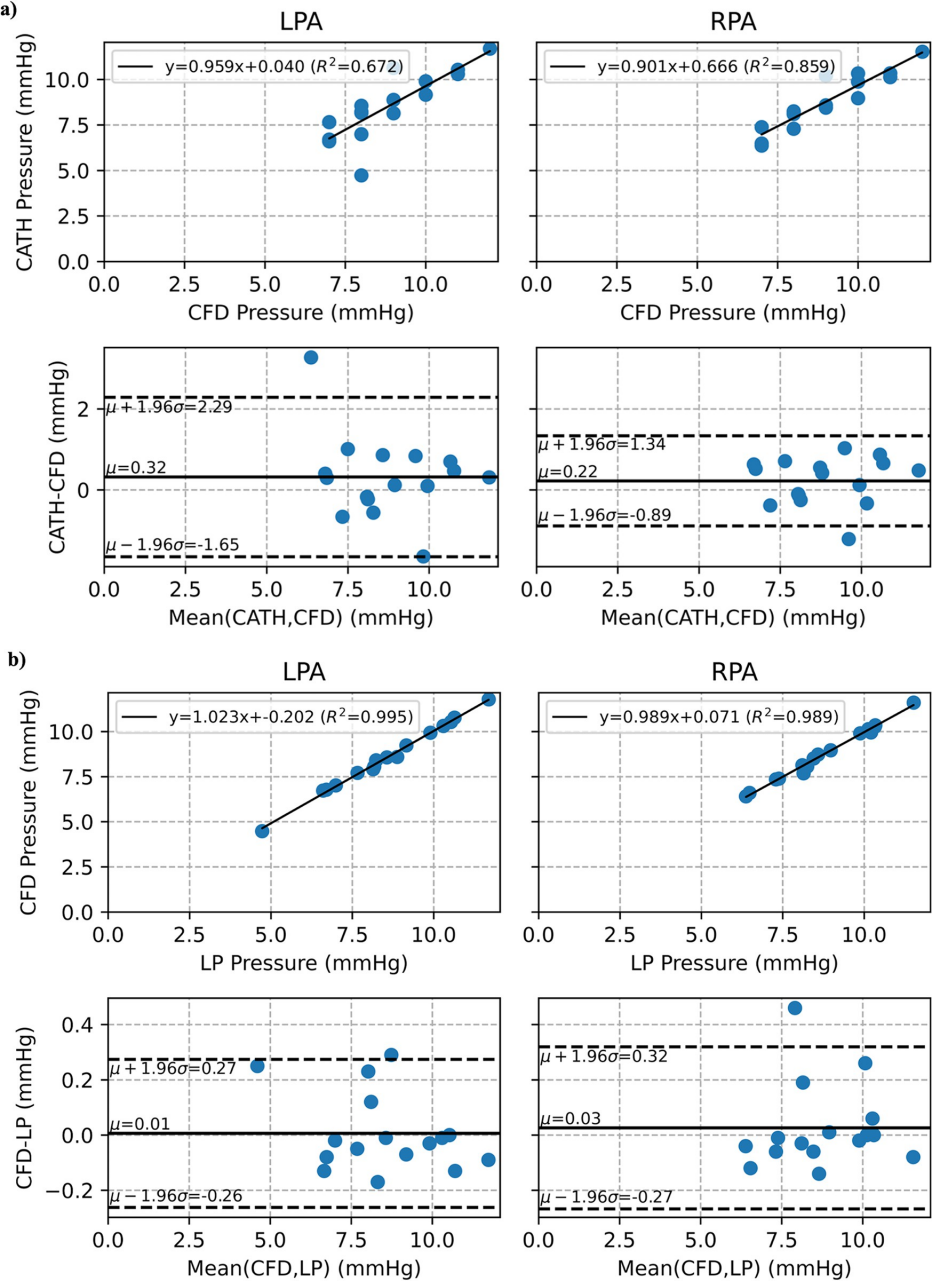
Mean outlet pressure comparison between (a) CATH and CFD, and (b) CFD and LP.

A similar comparison is made for the mean outlet pressure, where we again find excellent agreement between the CFD and LP models in both the LPA (CFD 8.555 ± 1.807 mmHg vs. LP 8.549 ± 1.853 mmHg; p-value = 0.992; ICC = 0.997 [0.99–1.00]) and the RPA (CFD 8.776 ± 1.506 mmHg vs. LP 8.750 ± 1.498 mmHg; p-value = 0.961; ICC = 0.995 [0.99–1.00]). However, we find a decrease in correlation when comparing the CFD and LP models’ mean outlet pressure to the CATH-measured mean outlet pressure (LPA 8.875 ± 1.544 mmHg, RPA 9.000 ± 1.549 mmHg), with the LPA pressure correlation being worse (CATH vs. CFD; p-value = 0.594; ICC = 0.805 [0.54–0.93] and CATH vs. LP; p-value = 0.592; ICC = 0.783 [0.49–0.92]) than the RPA pressure correlation (CATH vs. CFD; p-value = 0.682; ICC = 0.921 [0.79–0.97] and CATH vs. LP; p-value = 0.646; ICC = 0.934 [0.81–0.98]). Similar to outlet flow, Fig 5 shows the comparison between the mean LPA and RPA pressure relative to CATH-measured pressure and between the CFD and LP model, showing a slight decrease in agreement. Table 2 provides a summary of the flow and pressure results of both models, including the comparison to CMR flow and CATH pressure measurements.

**Table 2:** CMR, CATH, CFD, and LP data for the left and right lungs, comparing (a) mean indexed flow rates and (b) mean pressures.

*a)*		
**Flow Comparison**	***Q***_***LPA***_ (L/min/m^2^)	***Q***_***RPA***_ (L/min/m^2^)
**CMR**	0.971 ± 0.462	0.998 ± 0.284
**CFD**	0.980 ± 0.454	0.989 ± 0.279
**LP**	0.964 ± 0.447	0.977 ± 0.272
**Difference (CMR–CFD)**	-0.008 ± 0.043	0.009 ± 0.043
**Difference %**	-1.41% ± 5.15%	0.86% ± 4.12%
**Difference (CMR—LP)**	0.008 ± 0.042	0.021 ± 0.044
**Difference %**	0.21% ± 4.77%	1.93% ± 3.88%
**Difference (CFD—LP)**	0.02 ± 0.02	0.01 ± 0.02
**Difference %**	1.56% ± 1.74%	1.06% ± 2.01%
**ICC [95% CI] (CMR, CFD)**	0.996 [0.99–1.00]	0.988 [0.97–1.00]
**ICC [95% CI] (CMR, LP)**	0.996 [0.99–1.00]	0.986 [0.96–1.00]
**ICC [95% CI] (CFD, LP)**	0.999 [0.99–1.00]	0.997 [0.99–1.00]
**P-value (CMR, CFD)**	0.960	0.929
**P-value (CMR, LP)**	0.962	0.834
**P-value (CFD, LP)**	0.921	0.904
*b)*		
**Pressure Comparison**	***P***_***LPA***_ (mmHg)	***P***_***RPA***_ (mmHg)
**CATH**	8.875 ± 1.544	9.000 ± 1.549
**CFD**	8.555 ± 1.807	8.776 ± 1.506
**LP**	8.549 ± 1.853	8.750 ± 1.498
**Difference (CATH–CFD)**	0.320 ± 1.037	0.224 ± 0.586
**Difference %**	3.58% ± 12.62%	2.32% ± 6.56%
**Difference (CATH—LP)**	0.326 ± 1.114	0.250 ± 0.513
**Difference %**	3.64% ± 13.55%	2.64% ± 5.69%
**Difference (CFD–LP)**	0.01 ± 0.14	0.03 ± 0.15
**Difference %**	0.21% ± 2.01%	0.26% ± 1.85%
**ICC [95% CI] (CATH, CFD)**	0.805 [0.54–0.93]	0.921 [0.79–0.97]
**ICC [95% CI] (CATH, LP)**	0.783 [0.49–0.92]	0.934 [0.81–0.98]
**ICC [95% CI] (CFD, LP)**	0.997 [0.99–1.00]	0.995 [0.99–1.00]
**P-value (CATH, CFD)**	0.594	0.682
**P-value (CATH, LP)**	0.592	0.646
**P-value (CFD, LP)**	0.992	0.961

### 3.2. PVR estimation

**Fig 6:**
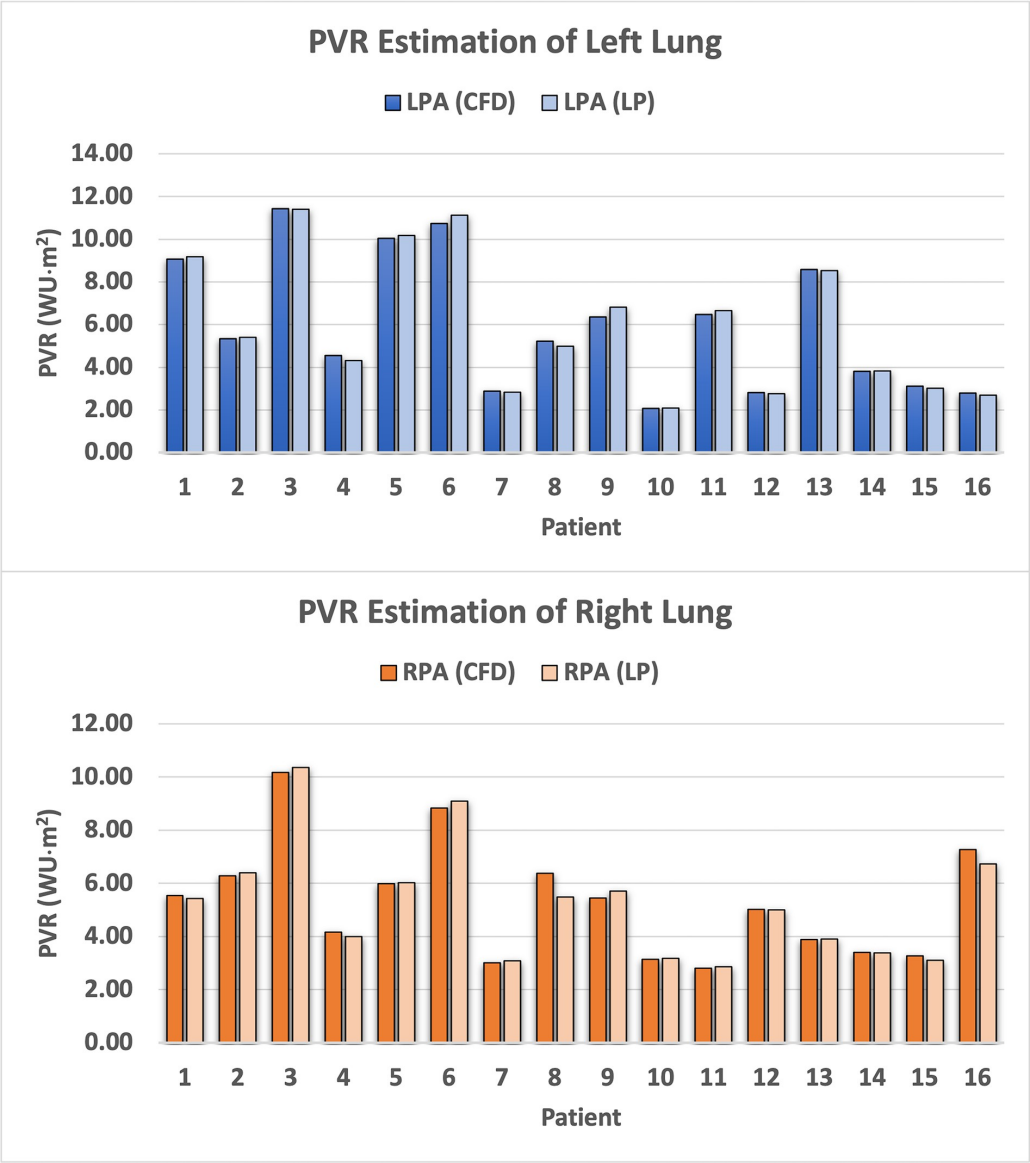
PVR estimates for the left and right lung of all 16 patients resulting from the PVR estimation pipeline.

The mean PVR estimates of the two models compare favorably as well, with the left lung estimates (CFD 5.957 ± 3.131 WU·m^2^ vs. LP 5.991 ± 3.218 WU·m^2^; p-value = 0.976; ICC = 0.998 [0.99–1.00]) and the right lung estimates (CFD 5.292 ± 2.159 WU·m^2^ vs. LP 5.237 ± 2.184 WU·m^2^; p-value = 0.943; ICC = 0.991 [0.98–1.00]) both showing good agreement between the two models, as shown in Table 3 and Fig 6. Relative to the CFD PVR estimates, the mean percent difference of the LP PVR estimates is 0.07% ± 3.11% for the left lung and 1.00% ± 4.70% for the right lung.

**Table 3:** Comparison of PVR estimates for the left and right lungs across all 16 patients.

PVR Comparison	LPA PVR (*WU*∙*m*^2^)	RPA PVR (*WU*∙*m*^2^)
Computed (*ΔP*_*CATH*_/*Q*_*CMR*_)	6.183 ± 3.240	5.290 ± 2.107
**CFD**	5.957 ± 3.131	5.292 ± 2.159
**LP**	5.991 ± 3.218	5.237 ± 2.184
**Difference (Computed–CFD)**	0.225 ± 0.371	-0.002 ± 0.363
**Difference %**	3.21% ± 5.35%	0.51% ± 6.52%
**Difference (Computed–LP)**	0.191 ± 0.282	0.054 ± 0.245
**Difference %**	3.35% ± 4.68%	1.71% ± 4.60%
**Difference (CFD–LP)**	-0.034 ± 0.187	0.056 ± 0.294
**Difference %**	0.07% ± 3.11%	1.00% ± 4.70%
**ICC [95% CI] (Computed, CFD)**	0.991 [0.97–1.0]	0.986 [0.96–1.0]
**ICC [95% CI] (Computed, LP)**	0.995 [0.98–1.0]	0.994 [0.98–1.0]
**ICC [95% CI] (CFD, LP)**	0.998 [0.99–1.0]	0.991 [0.98–1.0]
**P-value (Computed, CFD)**	0.843	0.998
**P-value (Computed, LP)**	0.868	0.944
**P-value (CFD, LP)**	0.976	0.943

### 3.3. PVR validation

The only available clinical metric for validation was the patient total PVR, which was computed from the CATH-based indexed pulmonary flow rate based on the Fick principle. We compared the indexed pulmonary flow rate measured by CMR and CATH and, as shown in Table 1, indicates that the CMR-measured indexed flow rate was significantly different from the CATH-based indexed flow rate (CMR 1.97 ± 0.56 L/min/m^2^ vs. CATH 2.73 ± 1.00 L/min/m^2^; p-value = 0.013; ICC = 0.209 [-0.15–0.58]). We then computed the total PVR from the optimized left and right lung PVR estimates and compared it to the CATH-derived total PVR. Both the CFD total PVR estimates (2.68 ± 1.19 WU·m^2^) and the LP total PVR estimates (2.68 ± 1.23 WU·m^2^) show poor agreement with the CATH-based total PVR (1.80 **±** 0.75 WU·m^2^; CATH vs. CFD; p-value = 0.019; ICC = 0.47 [-0.07–0.79]; CATH vs. LP; p-value = 0.022; ICC = 0.47 [-0.07–0.79]). The CFD total PVR estimates resulted in a relative mean difference of -56.10% ± 57.05% from the CATH-based total PVR, while the LP total PVR estimates yielded a relative mean difference of -55.09% ± 56.03%, indicating both models predict consistently higher values of PVR compared to the CATH-based PVR.

**Fig 7:**
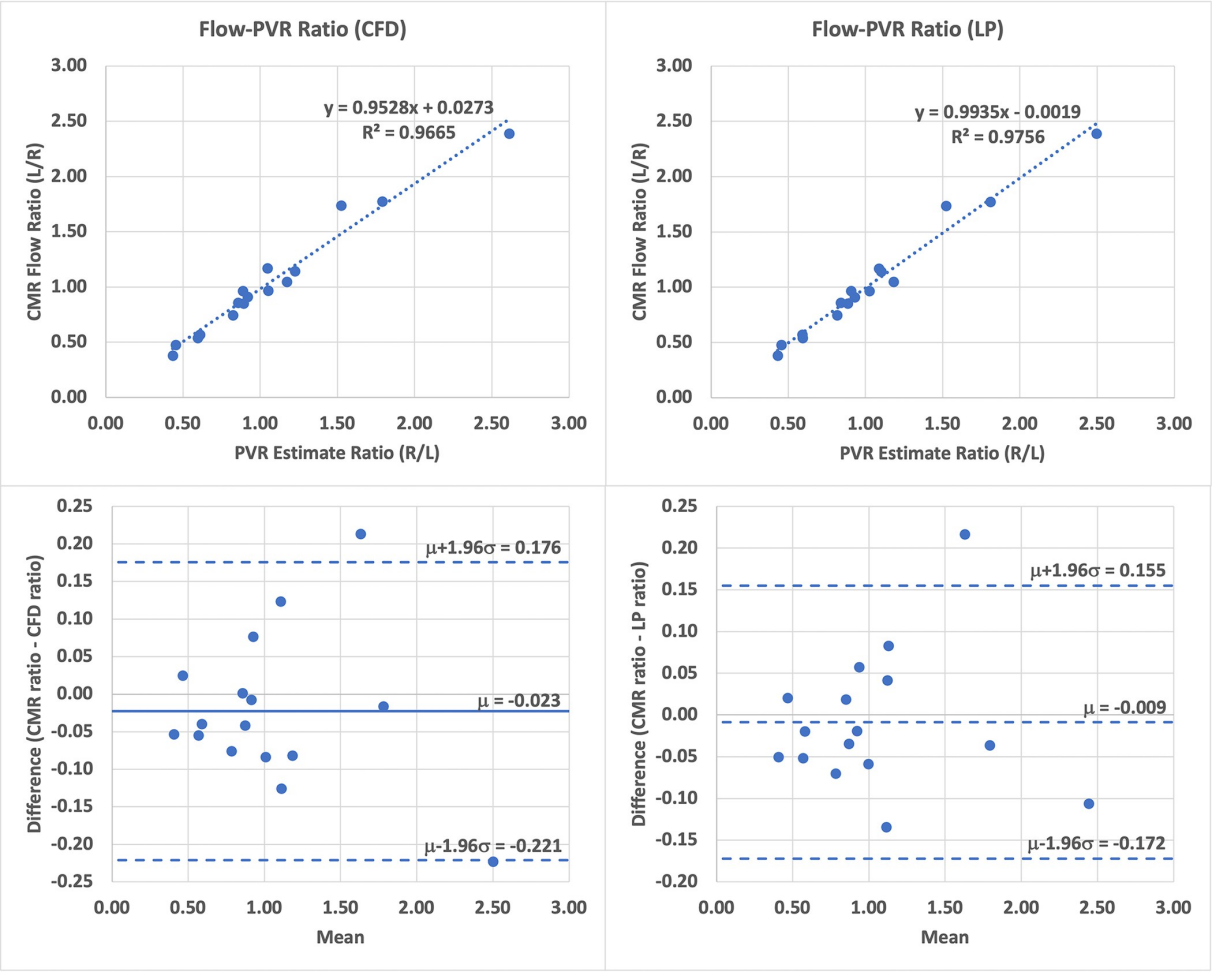
Right-left optimized PVR estimate ratios correlate well with left-right CMR flow ratios in both the CFD and LP model.

In addition to the total PVR, we compared the optimized PVR estimates to the left and right lung PVR estimated by computing the ratio of CATH pressure gradients and CMR outlet flows, i.e. $R_{L}=\frac{P_{SVC}-P_{LA}}{Q_{LPA}}$ and $R_{R}=\frac{P_{SVC}-P_{LA}}{Q_{RPA}}$. We found that the optimized PVR estimates agree very well with the computed resistance estimates, as seen in Table 3, with CFD estimates having relative mean differences of 3.21% ± 5.35% (LPA) and 0.51% ± 6.52% (RPA), while LP estimates show a relative mean difference of 3.35% ± 4.68% (LPA) and 1.71% ± 4.60% (RPA). Fig 7 further highlights this agreement by showing a strong correlation between the CMR flow ratios and the optimized PVR ratios.

### 3.4. Computational performance

The computational overhead between the CFD and LP models was compared to provide motivation for the use of the LP model. As expected, results show that single simulation processing time of the LP model is significantly shorter than that of the CFD model (CFD 595.53 s ± 233.75 s vs. LP 0.16 s ± 0.01 s). Given that one optimization run for a single patient takes approximately 100 simulations, this means the CFD optimization takes approximately 16 hours per patient (using 4 processors). This is compared to the LP model, which takes only about 16 seconds for a single optimization run. Thus, with the relatively simple models employed for this study, it is reasonable to at least begin the optimization with the LP model, and if needed, further refinement can be made with the CFD model.

## 4. Discussion

In this study, we explored an optimization pipeline for estimating PVR in patient-specific 3D models of the Glenn pathway and evaluated the feasibility of using a simplified 0D LP model to perform the same PVR estimation in a more computationally efficient manner. The first method creates a 3D model using CMR image segmentation and estimates the PVR of the left and right lung via 3D CFD simulations, while the second method uses an LP model to generalize the vasculature into a 0D electrical analogue and uses 0D simulations to estimate the PVRs. Previous studies have found LP models to be useful for predicting hemodynamic effects, particularly 1D models studying the hemodynamic effects of blood vessel elasticity [29–31]. Some of these models also incorporated 0D submodels for parts of the circulatory model, which are similar to our 0D LP model. Performing PVR estimation using the LP model results in significantly reduced computation time compared to the CFD model, with only a slight deviation in accuracy. The advantage of using a computational approach to predict the PVR in lieu of simply computing it via CATH pressure and CMR flow is the granularity of the resulting PVR data. We can not only estimate the PVR of the left and right lung separately, but also estimate the individual resistances along the Glenn pathway. For example, we can break down the estimated PVR into outlet resistance and the resistance of the individual vessels of the Glenn pathway (SVC, LPA, and RPA), as shown in Fig 3. This decomposition of PVR could be useful in future Fontan surgical planning when determining the optimal location for IVC anastomosis, given that the resistance of the individual vessels of the Glenn pathway can differ from one patient to another (Fig 1).

Across all 16 patients in this study, our optimization pipeline was able to accurately predict outlet flows relative to CMR flow, with both CFD and LP models generally predicting the mean outlet flows to within 2% on average, which is consistent with previous studies [32]. Pressure errors are slightly higher for both models, especially in the LPA, but still fall within an average of 5% of the CATH measured outlet pressure. It is possible this increased pressure error is because there is only a single comparative pressure value for the inlet and outlets, and the exact location of these CATH measurements is unknown. It is also possible that comparing measurements across multiple modalities, which were utilized at different times, results in a circuit that violates the conservation of mass/momentum. Therefore, optimizing experimental data to a model that ensures that mass/momentum is conserved is likely to result in some error. However, it also offers a correction to put the entire hemodynamic system into equilibrium with respect to Newton’s second law.

In general, both the CFD and LP models made similar PVR estimations, with the LP model estimation being within 1% of the CFD model estimation on average. The flow predictions of the LP model align well with the CFD model’s flow predictions, differing only by a mean of approximately 1%. Overall, the LP model performed similarly to the CFD model in terms of PVR estimation and flow/pressure errors which, combined with its significantly reduced computational overhead, provides an excellent starting point for PVR estimation, which could be used to initialize or tune boundary conditions for a subsequent CFD model, similar to the approach suggested in [33]. However, both models’ PVR estimations showed poor agreement with the clinically computed PVR, which is based on the Fick principle. Our clinical flow data indicates a significant discrepancy in pulmonary flow between CMR and CATH pulmonary flow measurements, similar to findings in related studies [16, 17]. This creates uncertainty about the accuracy of CATH-based PVR based on the Fick principle and makes it difficult to validate our PVR estimation pipeline. However, our PVR estimates do agree well with those computed via CATH pressure and CMR flow, which is the same input data for the optimization pipeline. This sets the stage for future validation studies and possibly expanding the optimization to include proximal/distal compliance which, when combined with resistance, presents identifiability issues.

There were several challenges and limitations for the current PVR estimation framework. First, our sample size was relatively small. Ideally, a larger patient cohort would enable more definitive conclusions. Second, the timing of the CMR and CATH measurements were inconsistent, with some patients having both exams on the same day, while others having months or even a year in between the two measurements. During this time difference between the CMR and CATH examinations, the patients’ physiology and hemodynamics may significantly change. Third, more validation is necessary, considering the only comparison to clinical data that is not used in the optimization procedure (and thus biased) is the PVR, which is itself problematic due to lack of agreement between Fick-based flow and CMR flow. Fourth, the CFD models assumed blood to be Newtonian, but it has been suggested that in some non-pulsatile, low velocity circulations like the Fontan circulation, the effects of non-Newtonian flow could be important to consider [34]. Therefore, the non-Newtonian effect on the results of our CFD simulations should be investigated. Finally, blood vessels in the CFD model are assumed to have rigid walls, thus compliance was not incorporated in the distal circulation. Adding compliance into the models, however, introduces identifiability issues by incorporating an additional parameter that can, along with resistance, have a redundant effect on pressure.

## 5. Conclusion

We developed a patient-specific in-silico PVR estimation pipeline, using both a 3D CFD model and a simplified 0D LP model, and tested it on 16 patients with Glenn physiology. Results showed that optimized PVR estimations resulted in accurate outlet flow and pressure when compared to CMR and CATH data. The CFD and LP models agree well on flow, pressure, and PVR estimates, while predicting a similar PVR to the computed PVR estimates from CATH and CMR data. However, both the computed PVR estimates and the optimized PVR estimates consistently overestimate clinical Fick-based PVR values, which we hypothesize to be the result of CATH overestimation of pulmonary flow in patients with Glenn physiology. Future efforts will allow for further validation of our PVR estimation pipeline and lay the groundwork for future virtual Fontan surgical simulation.
